# Uncertainty and depressive symptoms as mediators of quality of life in patients with heart failure

**DOI:** 10.1371/journal.pone.0205953

**Published:** 2018-11-14

**Authors:** Ting-Yu Chen, Chi-Wen Kao, Shu-Meng Cheng, Yue-Cune Chang

**Affiliations:** 1 Graduate Institute of Medical Sciences, National Defense Medical Center, Taipei, Taiwan; 2 Chung-Jen Junior College of Nursing, Health Sciences and Management, Chiayi, Taiwan; 3 Department of Nursing, Tri-Service General Hospital, Taipei, Taiwan; 4 School of Nursing, National Defense Medical Center, Taipei, Taiwan; 5 Division of Cardiology, Department of Internal Medicine, Tri-Service General Hospital, National Defense Medical Center, Taipei, Taiwan; 6 National Defense Medical Center, School of Medicine, Taipei, Taiwan; 7 Department of Mathematics, Tamkang University, Taipei, Taiwan; University of Birmingham, UNITED KINGDOM

## Abstract

Uncertainty in illness is regarded as a source of stress in many chronic diseases and is negatively related to health-related quality of life (HRQoL). However, studies on the relationship between uncertainty and HRQoL in patients with heart failure are limited. This study used Mishel’s theory of uncertainty in illness to investigate the mediating role of uncertainty in illness and depressive symptoms between symptom distress and HRQoL in patients with heart failure. This study used a cross-sectional correlation design. Participants were recruited by convenience sampling from outpatient services and medical wards of cardiology departments of a medical center in northern Taiwan. Data were collected for uncertainty, depressive symptoms, symptoms distress of heart failure, and HRQoL using self-report questionnaires. Demographics and clinical characteristics were analyzed with descriptive statistics. The mutual effects of disease characteristics, symptom distress, uncertainty in illness, depressive symptoms and HRQoL, as well as the overall model fitness, were analyzed by with structural equation modeling. We collected 147 qualified questionnaires. The mean score for the Mishel Uncertainty in Illness Scale for patients with heart failure was 73.5 (SD = 18.55); 65.3% of participants had a score of ≧13 on the Beck Depressive Inventory-II, indicating mild depression. Uncertainty, depressive symptoms, and HRQoL were directly related to symptom distress. Symptom distress and depressive symptoms were both mediators between uncertainty and depressive symptoms. Depressive symptoms also mediated emotional support and HRQoL. Uncertainty and depressive symptoms were important factors in the pathway between symptom distress and HRQoL for heart failure patients. We suggest providing heart failure patients with tailored interventions for effective self-management of symptoms based on Mishel’s theory of uncertainty in illness, which could help control disease symptoms, alleviate uncertainty and depression as well as improve HRQoL.

## Introduction

Heart failure (HF) is one of the significant global health issues and a serious health condition impacting many developing and developed countries, including Taiwan. It is estimated that over 5.1 million people in the United States have HF and over 650,000 patients are diagnosed with HF for the first time each year. The total medical costs for treating HF amount to more than US$3.92 billion [1]. The prevalence of HF is 0.4% to 6.0% in Asia, while values 6% was reported from Taiwan [2]. The cost of hospitalization was up to US$ 96 million in patients with HF in Taiwan [2]. The increasing prevalence of HF has resulted in an increased concern for HF as a clinical issue [3]. Due to the advancement of medical care, many HF patients are surviving longer; however, their prognosis remains poor and the 1-month re-hospitalization rate is approximately 17.1% [4].

The heart is pivotal for the circulation of blood. When the heart malfunctions, symptoms related to HF and short of the self-care capabilities impact not only the physical health of the patients but also their health-related quality of life (HRQoL) [5]. The varied and uncontrollable symptoms caused by HF may result in recurrent hospitalization and social isolation [6], which can lead to experience of uncertainty in illness [7]. HF patients frequently experience depression, which has been linked to increased risk of hospitalization and death [8]. Although uncertainty in illness is regarded as a source of stress in many chronic disease patients [9] and negatively related to HRQoL [10], studies on the relationship between uncertainty and HRQoL in patients with HF are limited. One study on uncertainty in illness and HRQoL in patients waiting for coronary artery bypass grafting surgery found that the lack of available information provided by health care professionals and the symptom distress caused by diseases during the waiting period for surgery may increase the uncertainty in illness and affect HRQoL [11]. Another longitudinal study on patients waiting for coronary angiography showed that uncertainty in illness was negatively related to HRQoL [12]. The study of Flemme et al. (2005) on patients with implanted cardioverter defibrillators revealed that uncertainty in illness was a key predictor of HRQoL [13].

Improving HRQoL for patients with cardiovascular disease by alleviating symptom distress and decreasing uncertainty in illness are important in disease management. However, few studies have investigated the relationship between uncertainty in illness and HRQoL in patients with HF. Moreover, no study has investigated the mediating effect of emotional factors such as uncertainty and depressive symptoms on HRQoL. Therefore, the purpose of this study was to investigate the mutual relationships of uncertainty, depressive symptoms and HRQoL, based on Mishel's Uncertainty in Illness Theory (UIT). This study had three aims: (1) to explore uncertainty, depressive symptoms and HRQoL in HF patients; (2) to examine the relationships of uncertainty, depressive symptoms and HRQoL through testing a conceptual model based on Mishel’s UIT; and (3) to identify the mediating role of uncertainty and depressive symptoms in the relationship between symptom distress and HRQoL.

## Theoretical framework

Michel’s UIT, proposed in 1988, is a theoretical framework for describing how an individual copes with a disease when unable to predict the outcome of an illness. Michel’s UIT consists of three principal themes: antecedents of uncertainty, appraisal of uncertainty and coping with uncertainty [14].

*Antecedents of uncertainty* refers to experiences that have occurred prior to the onset of an illness and affect a person’s thinking, such as perception of disease, exposure to pain, and prior experiences. This theme includes three components: stimuli frame, cognitive capacity, and structure providers. Stimuli frame is the personal perception of diseases and therapy-related events, which consists of symptom pattern, event familiarity and event congruence. Symptom pattern is the number, frequency, intensity, and duration of disease symptoms. If the symptom patterns have a high variability, the individual may perceive more uncertainty. Event familiarity means the individual familiarity with the scenario of disease and therapy-related events. The higher the familiarity, the less uncertainty the individual has about the therapy, care system, and the complexity of the disease. Event congruence is the consistency between the individual expectation of disease-related events and their actual experiences. Incongruence incurs unexpected uncertainty. Cognitive capacity and structure providers cause positive effects on stimuli frame. Cognitive capacity exhibits the individual capability of managing information. Structure providers disclose the resources assisting the individual to interpret stimuli frame, including education, social support and credible authority.

*Appraisal of uncertainty* refers to how a person evaluates an illness situation, which can be viewed as a danger or an opportunity. Danger is the probability of a negative outcome, while opportunity is the probability of a positive outcome.

‘*Coping with uncertainty’* refers to how the strategies individual employs to manage the uncertainties of disease. Coping with a danger appraisal is regarded as a responsive action to decrease uncertainty, which deals with the emotion produced by a danger appraisal. Coping with a situation which is appraised as an opportunity, involves direct actions that will maintain positive feelings and hope.

Uncertainty always exists in the disease trajectory. When the role of the individual is changed, the daily functions are limited, the social communication is terminated, and the life of the individual is in danger because of the disease, uncertainty will show up naturally [15]. Under these circumstances, the individual may employ problem-focused or emotion-focused strategies to adapt to the difficult situations in order to maintain their quality of life [10].

**Fig 1:**
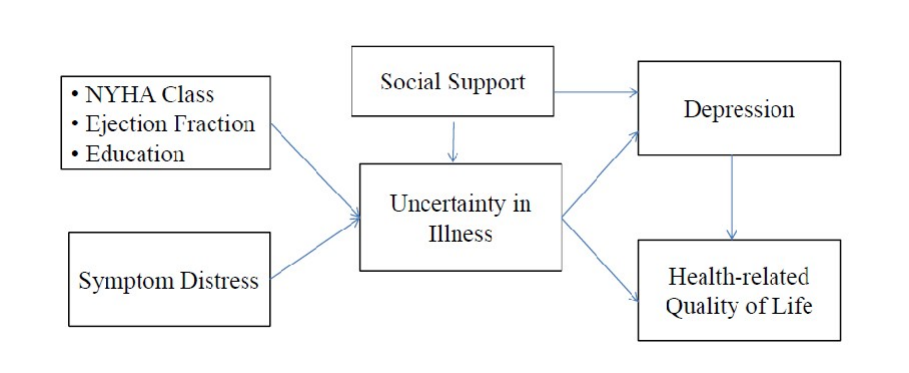
Conceptual Framework of this study. Conceptual Framework of uncertainty of illness and depressive symptoms in patients with heart failure. NHYA, New York Heart Association.

The framework for this study is based on Mishel’s UIT to investigate the mediating role of uncertainty and depressive symptoms between symptom distress and HRQoL in HF patients. The disease characteristics, symptom distress and social support are regarded as the antecedent factors of uncertainty. HRQoL refer to the outcomes after adaptation. The symptoms of heart failure such as fatigue, dyspnea, and weight gain may negatively affect the patient’s psychological well-being [16,17]. Previous studies found that deterioration of physical symptoms was significantly associated with depressive symptoms [18,19] and approximately 50% of HF patients have depressive symptoms [20]. Depression significantly increased all-cause mortality and was related to poor HRQoL in HF patients [21,22]. Because depression as a predictor of HRQoL for HF patients and significantly associated with symptom distress [23,24], we also added depressive symptoms into the model to identify the mediator role of depressive symptoms between symptom distress and HRQoL. The model for this study is shown in Fig 1

## Methods

We conducted a cross-sectional correlation study to examine the relationships of uncertainty in illness, depressive symptoms and HRQoL based on Mishel's UIT. Uncertainty, depressive symptoms, and HRQoL were measured by using Chinese versions of standardized instruments.

### Participants and procedure

We recruited study participants from a teaching hospital and medical center in northern Taiwan with convenience sampling. Patients diagnosed with HF were included if they met the following criteria: (1) older than 20 years, (2) with New York Heart Association (NYHA) Functional Class Ⅰ to Ⅳ, (3) without receiving heart transplant, permanent pacemaker or an implantable cardioverter defibrillator, (4) without addiction to drugs or alcohol, (5) not using antidepressants, and (6) without preexisting psychiatric diagnosis or cognitive impairment. The study was approved by the Institutional Review Board of the Tri-Service General Hospital National Defense Medical Centre. Prior to conducting the survey, patients were informed about the research purpose and procedure and were assured of anonymity. Written informed consent was obtained from all participants. A total of 147 HF patients participated in this study from January to June 2012. The estimates of power analysis revealed that the sample size provided the statistical power of 0.82 with the effect size at 0.15 and the significance (α) at 0.05 [25].

### Instruments

#### Personal characteristics questionnaire

We designed a personal characteristics questionnaire to collect data regarding the demographics and health characteristics of the participants.

#### Mishel uncertainty in illness scale

Patients’ uncertainty of disease was measured with the Chinese version of the Mishel Uncertainty in Illness Scale (MUIS), which consists of 25 items rated on a 5-point Likert scale from "strongly disagree" to "strongly agree". The responses represent the patient’s perception of uncertainty about their symptoms, diagnosis, treatment, relationship with caregivers, and prognosis. There are 15 items related to factors of unpredictability, and 10 items to factors of multi-attributed ambiguity. A higher score indicates greater uncertainty. Cronbach’s α for the total scale was 0.87, and for the unpredictability subscale and the multi-attributed ambiguity subscale was 0.85 and 0.66, respectively [26]. In this study, Cronbach’s α for the total scale was 0.95, and for the unpredictability subscale and the multi-attributed ambiguity subscale was 0.94 and 0.86, respectively.

#### Beck depression inventory: Second Edition

The Chinese version of the Beck Depression Inventory- Second Edition (BDI-Ⅱ) is a 21-item self-administrated scale to assess the symptoms of depression. There are four symptom statements for each item. Responses to the item are rated from 0 to 3. The lower scores indicate less severity of depressive symptoms. A total score between 0 and 13 indicates a normal mood, between 14 and 19 indicates a mild level of depressive symptoms, between 20 and 28 indicates a moderate level of depressive symptoms, and between 26 and 63 indicates a severe level of depressive symptoms. The original scale had good convergent validity and discriminant validity, and the Cronbach’s α was from 0.86 to 0.88 [27]. The Chinese version of the BDI-II had an adequate internal consistency when tested in patients with end-stage renal disease (Cronbach’s α = .82) [28]. In this study, the Cronbach’s α was 0.95.

#### Heart failure symptom distress scale

Based on guidelines for the management of HF developed by the American College of Cardiology Foundation (ACCF) and the American Heart Association (AHA) as well as other studies [29–32], we designed a Heart Failure Symptom Distress Scale (HFSDS) to evaluate patient’s symptom distress resulting from HF. The HFSDS assesses 17 symptoms of HF: fatigue, weakness, head heavy, dyspnea on rapid exertion, weight gain, dry cough, swelling in the legs or ankles, orthopnea, palpitations, dizziness, syncope, memory decline, chest pain, insomnia, nausea, poor appetite and inability to concentrate. Responses to the items are rated on 6-point Likert scale from 0 = "no suffering from symptoms" to 5 = "severe suffering from symptoms". A higher score reflects greater symptom distress. In this study, the content validity index was 0.98 evaluated by five cardiovascular disease experts, and the Cronbach’s α for the total scale was 0.92.

#### Social support scale

Social support was measured by the Social Support Scale (SSS), which was modified from the Inventory of Social Support Behaviors by the authors [33]. SSS is a 19-item self-report scale rated with a 4-point Likert-type format. It contains four subscales: emotional support, informational support, instrumental support, and appraisal support. A higher the score indicates a greater perception of available support. Five experts proficient in cardiology examined the content validity of the SSS. The content validity index for the total scale was 0.91. The Cronbach’s α for the total scale was 0.96, and for the emotional support subscale, informational support subscale, instrumental support subscale, and appraisal support subscale was 0.94, 0.83, 0.92, and 0.92, respectively [34].

#### Minnesota living with heart failure questionnaire

Participants’ HRQoL was measured with the Chinese version of the Minnesota Living with Heart Failure Questionnaire (MLHFQ), which is a 21-item scale to assess patient’s perception of the impact of HF on the physical, psychological, and socioeconomic aspects of their life. The MLHFQ is a 6-point Likert scale from 0 to 5. A higher score reflects a worse HRQoL. The Chinese version of the MLHFQ has been identified to have appropriate construct validity and internal consistency reliability. Cronbach’s α for the total scale was 0.94 [35]. In this study, the Cronbach’s α for the total scale was 0.95.

### Statistical analysis

Data were analyzed with the SPSS 18.0 and AMOS 8.0 statistical software packages. We analyzed demographic and health characteristics of participants through using the mean, standard deviation (SD), frequency and percentage. AMOS was used for SEM analysis. Left ventricular ejection fraction (LVEF), NYHA Class, education, symptom distress, social support and depressive symptoms were set as measured variables, while uncertainty in illness and HRQoL were set as latent variables. Modification indices (Wald test) were used to find a parsimonious model [36]. Goodness-of-fit tests were used to examine whether the research model fit well. The fit of the model was determined by using several methods including normalized Chi-square < 3 [37], goodness-of-fit index (GFI)>0.90, adjusted goodness-of-fit index (AGFI)>0.90 [38], comparative fit index (CFI)> 0.90 [39], and root mean squares error of approximation (RMSEA)<0.1 [40]. Furthermore, Sobel’s test was applied to identify whether both uncertainty and depressive symptoms were mediators. If we obtained a Z-value> 1.96, then the mediating effect was considered significant [41].

## Results

### Participant characteristics

A total of 147 HF patients participated in this study (male = 80, female = 67). The mean age was 71±13.29 years. Most of participants had completed elementary school (41%), and were unemployed (76.9%) and married (76.2%). Seventy participants were outpatients (47.6%) and 77 were inpatients (52.4%). Participants’ mean LVEF was 46.42% (SD = 17.20, range 10-80). Most proportion of the participants were in NYHA Class II (32.0%), followed by 31.3% in class III, 7.7% in Class IV and 9.5% in Class I. Participants’ characteristics are shown in (Table 1)。<}0{>SSSS outpatients (47.6%) and 77 were inpatients (52.4%). Participants’ mean LVEF was 46.42% (SD = 17.20, range 10–80). Most proportion of the participants were in NYHA Class II (32.0%), followed by 31.3% in class III, 7.7% in Class IV and 9.5% in Class I. Participants’ characteristics are shown in Table 1.

**Table 1:** Characteristics of participants (N = 147).

Variable	n (%)	Mean (SD)
Age		71.04 (13.29)
Gender		
Male	80 (54.4%)	
Female	67 (45.6%)	
Employment status		
Unemployed	113 (76.9%)	
Employed	34 (23.1%)	
Education		
None	23 (15.6%)	
Elementary school	60 (40.8%)	
Junior high school	28 (19.2%)	
Senior high school	22 (15.0%)	
University/college degree	11 (7.5%)	
Master degree	3 (2.0%)	
Marital status		
Not married	6 (4.1%)	
Married	12 (76.2%)	
Divorced	2 (1.4%)	
Widow	25 (17.0%)	
Separated	2 (1.4%)	
Treatment		
Outpatient	70(47.6%)	
Inpatient	77(52.4%)	
NYHA Class		
Class Ι	14 (9.5%)	
Class Ⅱ	47 (32.0%)	
Class Ⅲ	46 (31.3%)	
Class Ⅵ	40 (27.2%)	
Left ventricle ejection fraction		46.42 (17.20)
Comorbidities		
Diabetes	55(37.4%)	
Renal disease	23(15.6%)	
Chronic obstructive pulmonary disease	23(15.6%)	

Note: SD, Standard deviation; NYHA Class, New York Heart Association Functional Classification

### Descriptive statistics of the measures

The mean, standard deviation, and score range of measures are presented in Table 2. The mean score of the HFSDS reported by the participants was 25.0 (SD = 13.25), indicating that participants had a low level of distress from disease symptoms. The highest distress mean scores among symptoms were for dyspnea on exertion (mean = 3.88, SD = 1.30), dyspnea while lying down (mean = 2.78, SD = 1.84) and sudden awaking due to dyspnea while sleeping (mean = 2.72, SD = 1.86). The mean score for the MUIS was 73.5 (SD = 18.55), indicating that participants had a moderate level of uncertainty in illness. The highest subscale score was unpredictability (mean = 52.06, SD = 13.73). The mean score of the SSS was 54.49 (SD = 11.51), suggesting that participants perceived a moderate level of social support. The highest subscale score was emotional support (mean = 16.03, SD = 3.05). The mean score of BDI-II was 19.42 (SD = 11.29), meaning that the participants had mild depressive symptoms. The mean score of the MLHFQ for participants was 54.41 (SD = 21.24), suggesting that participants had poor quality of life. The highest mean score between two domains was physical domain (mean = 25.44, SD = 11.30), indicating that participants perceived more impact from HF on the physical domain than on the psychosocial domain.

**Table 2:** Scores of measurements.

Measurements	Mean (SD)	Range
Heart Failure Symptom Distress Scale	25.03 (13.25)	0–85
Mishel Uncertainty in Illness Scale		
Total score	73.51 (18.55)	25–125
Unpredictability subscale score	52.06 (13.73)	15–75
Multi-attributed ambiguity subscale score	52.06 (13.73)	10–50
Social Support Scale		
Total score	54.14 (11.31)	19–76
Emotional support subscale score	13.58 (3.65)	5–20
Information support subscale score	16.03 (3.05)	6–24
Instrumental support subscale score	12.88 (3.08)	4–16
Appraisal support subscale score	11.65 (2.89)	4–16
BDI-Π	19.42 (11.29)	0–63
MLHFQ	54.41 (21.24)	0–105

Note: SD, standard deviation; BDI-II, Beck Depression Inventory: Second Edition; MLHFQ, Minnesota living with heart failure questionnaire

### Model testing and mediation model analysis

**Fig 2:**
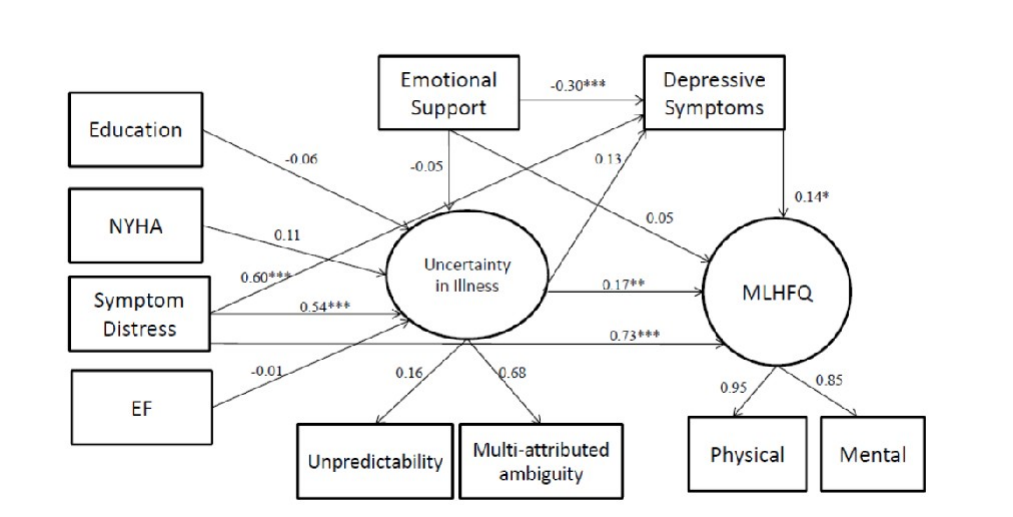
The original model. The original model of uncertainty to predict HRQoL for heart failure patients. NYHA, New York Heart Association functional class, EF, ejection fraction. *p-value < 0.05, **p-value<0.01, ***p-value < 0.001.

**Fig 3:**
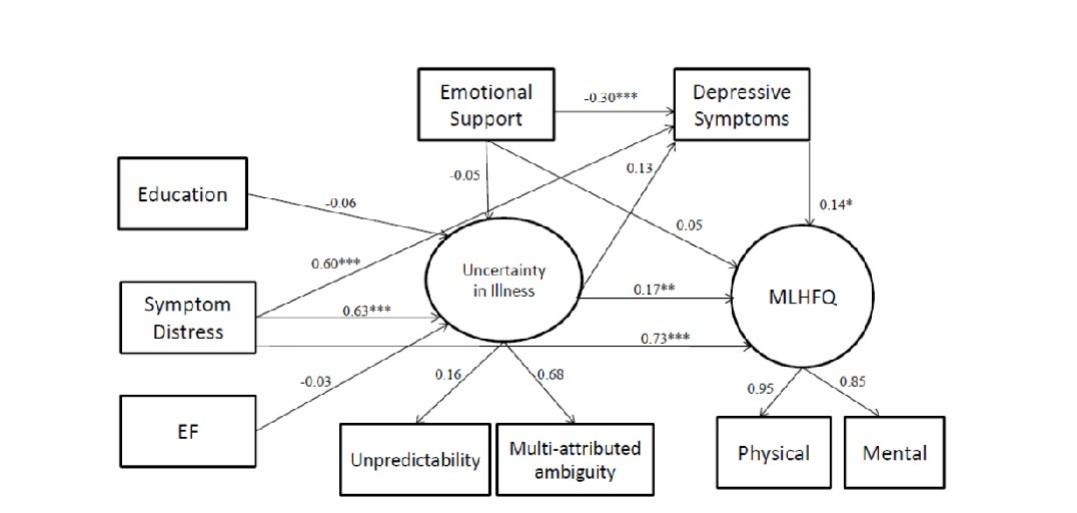
The modified model. The modified model of uncertainty to predict HRQoL for heart failure patients. *p-value < 0.05, **p-value<0.01, ***p-value < 0.001.

Our original model, based on Mishel's UIT, set the measured variables as education, NYHA class, LVEF, symptom distress, emotional support, and depressive symptoms. Uncertainty in illness and HRQoL were the latent variables. The original model had good fit indexes of *χ*^2^/df = 2.44, GFI = 0.92, AGFI = 0.84, CFI = 0.96, RMSEA = 0.10. However, the path coefficients between LVEF, NYHA class, education, and uncertainty were not significant (Table 3)(Fig 2). Therefore, we removed NYHA class, based on modification indexes. The modified model had better fitness indexes: *χ*^2^/df = 2.60, GFI = 0.93, AGFI = 0.85, CFI = 0.96, and RMSEA = 0.10 (Table 3) (Fig 3). Fifty-six percent of the variance in participants’ uncertainty was explained by symptom distress, emotional support and depressive symptoms. Eighty-nine percent of the variance in participants’ HRQoL was explained by symptom distress, emotional support, uncertainty and depressive symptoms. The results of Sobel's test showed that both uncertainty (Z value = 3.12; *p* value < 0.01) and depressive symptoms (Z value = 2.38; p value < 0.01) were mediators between symptom distress and HRQoL. In addition, the variable of depressive symptoms was also a mediator between emotional support and HRQoL (Z value = -2.25; p value < 0.01).

**Table 3:** The model fit indexes of structural equation models.

Model Fit Indexes							
Model	χ^2^	df	χ^2^/df	GFI	AGFI	CFI	RMSEA
Original	68.42	28	2.44	0.92	0.84	0.96	0.10
Modified	54.74	21	2.61	0.93	0.85	0.96	0.10

Note: χ^2^, Chi-square test value; df, degrees of freedom; GFI, goodness-of-fit index; AGFI, adjusted goodness-of- fit index; CFI, comparative fit index; RMSEA, root mean squares error of approximation.

Symptom distress had direct effects on uncertainty (β = 0.63, p < .001), depressive symptoms (β = 0.60, p < .001) and HRQoL (β = 0.73, p < .001) as well as indirect effects on HRQoL through uncertainty and depressive symptoms. Emotional support had direct effects on depressive symptoms (β = -0.30, p < .001), as well as indirect effects on HRQoL through depressive symptoms. The model in our study expressed more HF symptoms led to more uncertainty and depressive symptoms, which further bring about worse HRQoL. Otherwise, higher social support resulted in less depressive symptoms and then led to better HRQoL. The total, direct and indirect effects were shown in Table 4.

**Table 4:** Direct, indirect and total effects.

	Uncertainty			Depressive symptoms			HRQoL		
	IE	DE	TE	IE	DE	TE	IE	DE	TE
Symptom distress	-	.63***	.63***	.08	.60***	.68	0.11***	.73***	.83
Emotional support	-	-.05	-.05	-	-.30***	-.30***	-	.05	.05
Uncertainty	-	-	-	-	.13	.13	-	.17**	.17**
Depressive symptoms	-	-	-	-	-	-	-	.14*	.14*

Note: HRQoL, Health related quality of life; IE, Indirect Effect; DE, Direct Effect; TE, Total Effect

*p-value < 0.05

**p-value<0.01

***p-value < 0.001

## Discussion

The causal mechanism between symptom distress, uncertainty, depressive symptoms and HRQoL is rarely determined in patients with HF. The purpose of this study was to examine whether uncertainty and depressive symptoms are able to influence the relationship between symptom distress and HRQoL. Therefore, we conducted structural equation modeling analyses to test three hypotheses: (1) symptom distress, uncertainty, depressive symptoms and HRQoL were closely correlated in the structural equation model; (2) symptom distress showed a significant positive correlation with uncertainty and depressive symptoms, and a significant negative relationship with HRQoL, as well as an indirect influence on HRQoL through uncertainty and depressive symptoms; and (3) uncertainty and depressive symptoms mediated the relationship between symptom distress and HRQoL in HF patients.

The study results supported the first and second hypotheses. The symptom distress was significantly positively related to uncertainty and depressive symptoms, and negatively associated with HRQoL in patients with HF. Again, the symptom distress was able to indirectly influence HRQoL through uncertainty and depressive symptoms. The results are similar to the findings of a previous study showing that HF patients had moderate uncertainty (mean = 71.41, SD = 9.56) due to complex symptoms [42]. In the research of Kang also pointed out that atrial fibrillation patients with greater symptoms had more uncertainty (β = 0.21, p < 0.05) [43]. Bailey et al. [44] conducted a cross-sectional study in chronic Hepatitis C patients and showed that uncertainty was negatively associated with HRQoL. Because HF comprises a series of complex clinical symptoms, the symptoms are not only difficult to be treated, but also interfere with patients’ physical, psychological and social functions, and even HRQoL [45].

Our study also found that approximately 65.3% of HF patients scored over 13 on the BDI-II. This finding indicates that most HF patients had depressive symptoms, which is similar to a study showing 50% of HF patients suffer from depressive symptoms [22]. The findings of our study also show a positive relationship between symptom distress and depressive symptoms; indicating that patients with fewer symptoms had less depressive symptoms in our study. The findings are consistent with previous studies revealing a positive relationship between symptom distress and depressive symptoms [19,46]. Depressive symptoms are also a significant predictor of HRQoL in patients with HF [23,24]. In our study, a positive relationship between the score of BDI-II and the score of MLHFQ was detected. The outcomes in our study mean that patients with more depressive symptoms had a poor HRQoL. The HF patients with depressive symptoms have a poor adherence to medical treatments and healthy lifestyle, which lead to a decline in health status and affected their HRQOL [23,24, 46].

We also found a negative association between emotional support and depressive symptoms. This result is consistent with prior studies showing that HF patients with better emotional support had lower degree of depression. Social support is a buffering factor that can defend against such harmful events from HF or its treatments [47,48].

We found a mediator effect of uncertainty in the relationship between symptom distress and HRQoL. Mishel (1988) believed that uncertainty was generated when individuals were unclear about the disease treatment and outcome, and could not engage in proper sorting or compiling of information [49]. HF patients often face a load of symptoms such as fatigue, breathlessness, and functional impairment [19,50]. When the patient lacks information about the disease and feels a loss of control over the disease, uncertainty tends to increase as a result of its complex nature [12,43]. Jiang et al. exposed that uncertainty management intervention has a definite effect on decreasing emotional symptoms and improving HRQoL in chronic obstructive pulmonary disease patients [51]. The results showed that patients received uncertainty management intervention had significant lower degree of uncertainty than that of patients without receiving intervention. Again, the decrease of uncertainty resulted in the improvement of emotional symptoms and HRQoL [51].

Our findings demonstrated depressive symptom was the mediator between symptom distress and HRQoL, and also the mediator between emotional support and HRQoL. Hallas et al. indicated that HF patients with depression have more negative beliefs about their disease and more negative coping behaviors to cause poor quality of life [52]. Depressive symptoms will adversely influence HF patients’ adherence to the treatment regimen [46, 53]. The consequence will impair patients’ physical functioning, increase symptom distress, and worse HRQoL [8, 54]. Noroozi et al. reported the effectiveness of group cognitive behavior therapy on depression and quality of life in women with Type 2 diabetes [55]. They found that patients receiving cognitive behavior therapy had significant improvement on depression and HRQoL than those of the patients without receiving this intervention. Hence, the reduction of depression led to the improvement of mental condition and HRQoL [55].

## Limitations

This study had some limitations. First, this was a cross-sectional design; a future study using a longitudinal research design is recommended to fully understand changes in patient's uncertainty in illness and quality of life on the whole course of a disease. Second, we only recruited patients from one medical center, which limits generalization of the findings. Future research should be conducted in multiple centers to make the research findings more representative of a larger population. Finally, our study framework is to take the educational level of the study subjects as the “cognitive capacity” mentioned in the theory of uncertainty in illness. We did not use additional questionnaires to test the cognitive capacity of the study subjects. We suggest that if future studies are based on Mishel’s theory of uncertainty in illness, a cognitive capacity test could be added in the cognitive capacity section of the theory.

## Conclusions and implications

This study is the first study based on the Mishel’s UIT to indicate the mediation effect of uncertainty and depressive symptoms on the relationship between symptom distress and HRQoL in HF patients. These findings suggest that decreasing uncertainty and depressive symptoms could enhance HF patients’ HRQoL. Based on Mishel’s UIT, nurses and healthcare professionals should design systematic interventions to help HF patients control disease symptoms, alleviate or reduce uncertainty and depression, and improve their HRQoL.

## Supporting information

S1 STROBE Checklist (PDF)
