# Correction: Uncertainty and depressive symptoms as mediators of quality of life in patients with heart failure

**DOI:** 10.1371/journal.pone.0214825

**Published:** 2019-03-28

**Authors:** Ting-Yu Chen, Chi-Wen Kao, Shu-Meng Cheng, Yue-Cune Chang

Authors Ting-Yu Chen and Chi-Wen Kao are incorrectly noted as equal contributors to this work. The full, correct byline is as follows:

Ting-Yu Chen, Chi-Wen Kao*, Shu-Meng Cheng‡, Yue-Cune Chang‡.

‡ These authors contributed equally to this work

* Corresponding author
